# Cognitive-Cognitive Dual-task in aging: A cross-sectional online study

**DOI:** 10.1371/journal.pone.0302152

**Published:** 2024-06-07

**Authors:** Giulio Contemori, Maria Silvia Saccani, Mario Bonato

**Affiliations:** 1 Department of General Psychology, University of Padova, Padova, Italy; 2 Padova Neuroscience Center, Padova, Italy; Universidade Federal do Para, BRAZIL

## Abstract

The prevalence of neurodegenerative disorders, particularly dementia, is on the rise across many countries worldwide. This negative trend calls for improving our understanding of cognitive aging. While motor-cognitive dual-task approaches have already been proven valuable for clinical diagnosis, comparatively less research is available on the application of Cognitive-Cognitive Dual-Tasking (CCDT), across several cognitive domains. Moreover, there is limited understanding about how healthy aging affects performance in such dual-tasks in the general population. CCDT entails engaging individuals in multiple cognitive tasks simultaneously and holds promise for remote e-Health interventions. In this cross-sectional study, our objective was to evaluate the suitability of a newly developed, self-administered, online tool for examining age-related differences in memory performance under dual-tasking. 337 healthy adults aged 50–90 underwent a visual memory test (Memo) under both single and dual-task conditions (attend to auditory letters). Additional measures included questionnaires on subjective memory complaints (MAC-Q), on cognitive reserve (CR), and a cognitive screening (auto-GEMS). As expected, the accuracy of visual memory performance exhibited a negative correlation with age and MAC-Q, and a positive correlation with CR and auto-GEMS scores. Dual-tasking significantly impaired performance, and its detrimental effect decreased with increasing age. Furthermore, the protective effect of cognitive reserve diminished with advancing age. These findings suggest that the commonly observed age-related increase in dual-task costs is not universally applicable across all tasks and cognitive domains. With further refinement, a longitudinal implementation of this approach may assist in identifying individuals with a distinct cognitive trajectory and potentially at a higher risk of developing cognitive decline.

## Introduction

In a healthy adult brain, information processing capacity is constrained [1]. This limitation impacts the simultaneous handling of multiple tasks, revealing the capacity boundaries of human cognition. McIsaac and colleagues define dual-tasking as the concurrent performance of two independent tasks with distinct goals [1]. The dual-task (DT) literature primarily explores how one task interferes with another when performed concurrently, often leading to a decline in performance in either one or both tasks. The taxonomy of DT is intricate, varying in task types, stimulus frequency, and response frequency. When focusing on task nature dual-tasking can be broadly classified into two main categories: Motor-Cognitive Dual-Tasking (MCDT), involving a motor task and a cognitive task, and Cognitive-Cognitive Dual-Tasking (CCDT), involving two concurrent cognitive tasks. Esmaeili Bijarsari (2021) [2] and McIsaac (2015) [1] have conducted comprehensive reviews on the characteristics of dual-tasking and proposed a taxonomy.

This article specifically focuses on CCDT, incorporating both a visual memory test and a sustained attention test with auditory letters. In a group of healthy adults aged 50–90 we investigated CCDT interference in relation with self-administered questionnaires and tests related to overall cognitive health and subjective memory impairment. The goal is to gain a better understanding of the trajectory of mnemonic decline in healthy older adults. In this scenario, the use of the dual task exposes the capacity limits resulting from aging, even in those with high cognitive reserves who would typically perform as well as younger individuals in single tasks.

Questions remain about how age impacts performance in dual-task situations. For example, while older adults show greater difficulty compared to younger adults in dual motor-cognitive tasks [3], findings for the CCDT are inconsistent [4–8]. Understanding the impact of aging upon cognition is not trivial as cognitive functions vary greatly in their lifelong trajectories. Fluid intelligence, for instance, peaks around the age of 20, to then gradually decline. On the contrary, crystallized intelligence improves until late adulthood [9]. Within this context it remains unclear whether DT performance is to be considered part of executive functions or, rather, domain-independent [10–12]. Global cognitive performance experiences a more noticeable decline only after reaching the late seventies [13, 14]. This decline may be attributed to age-related neurological deterioration, or to the impact of other medical conditions that adversely affect cognition. Within the same age cohort, some individuals maintain normal cognition, while others may exhibit an early, sudden decline, namely a deviation from the typical trajectory of healthy aging which potentially indicates a prodromal pathological state [15].

In the DT utilized in this study, the primary mnestic task required forced-choice image recognition (objects) [5] and was designed to prioritize familiarity over recollection. These two aspects tends to dissociate in clinical population as only familiarity-based recognition remains relatively stable across healthy aging, ensuring comparable task difficulty between young and old individuals [16]. Conversely, individuals with Mild Cognitive Impairment (MCI) or early Alzheimer’s disease typically perform poorly when recognition relies on familiarity [17–20].

In a previous study [5], we observed that a sustained attention task, although easy even for older participants, effectively modulated visuo-mnestic performance. In the present study, our primary goal was to extend earlier observations by integrating both objective and subjective measurements to comprehensively assess cognitive performance. Building upon previous findings, our objective was to outline a novel approach for measuring cognitive trajectories in aging.

Comprehending which cognitive aspects remain stable in healthy older adults but decline first in pathological aging is crucial for establishing a differential diagnosis of cognitive impairment [21]. This performance decline is more pronounced in individuals with neurodegenerative diseases, making the clinical application of DT a promising area of research [22, 23]. Specifically, attention and memory remain relatively stable in healthy aging but are significantly affected by pathological aging [24]. Combining the two cognitive functions might therefore be a particularly appropriate DT approach.

Several advancements have been made in predicting the transition from mild cognitive impairment (MCI) to dementia [25]. Individuals progressing to MCI typically experience initial clinical symptoms related to short-term memory issues, followed by declines in other cognitive domains [26]. Due to varying levels of cognitive reserve among individuals, early-stage AD impairment may be subtle and differ widely [27]. Clinicians need to select appropriate, sensitive tests to detect early decline, considering patients’ coping strategies and cognitive reserve [27]. One common approach involves looking for signs of decline through longitudinal testing, while another involves using difficult tasks to challenge the implementation of compensatory strategies. Adding a secondary task can be more demanding for persons with prodromal MCI compared to healthy individuals [1]. While Motor-Cognitive Dual-Tasking (MCDT) are largely utilized for this aim and follow a more common standard, there is no standardized method for conducting Cognitive-Cognitive Dual-Tasking in clinical practice [2]. A typical combination in MCDT involves concurrently performing a working memory task along with a walking task, such as the "timed Up & Go" task, which requires rising from a chair and walking a short distance while counting backward [28, 29]. Key common variables in MCDT that predict cognitive deficits include gait speed, speed cost, and the number of words produced during the dual-task [30]. MCDT tests have limited applications since they assess only one cognitive function at a time and may not be suitable for conditions lacking obvious motor symptoms, such as multiple sclerosis and prodromal MCI [31]. In contrast, CCDT overcomes these limitations inherent in MCDT [32]. By incorporating tasks from various cognitive domains—attention, memory, working memory, and executive functions—CCDT provides more comprehensive insights than assessing these domains individually alongside a motor task. Combining multiple cognitive tasks is likely to enhance sensitivity, surpassing the information obtained through MCDT [5, 31, 33].

When planning our study, our goal was to integrate a task that retained the predictive value of MCDT while leveraging the strengths of CCDT. To achieve this, we structured our CCDT similarly to clinical MCDT. This similarity is crucial because the predictive value of MCDT is often associated with sustained attentional engagement, lacking in standard dual tasks manipulating or eliciting responses on a trial-by-trial basis.

Consider the Psychological Refractory Period (PRP) task, one of the most well-known CCDTs [34]. In this task, participants handle two tasks on a trial-by-trial basis. The psychological refractory period denotes the interval during which the secondary task is affected by interference from the primary task. This setup assesses response selection, an executive component. The PRP paradigm differs fundamentally from the MCDT paradigm, where tasks occur simultaneously, and the motor task is performed continuously while the cognitive task is intermittent (i.e., backward counting). Our dual task (DT) adopts a comparable approach, merging a sustained attention task with intermittent responses and a memory task that involves continuous memorization with delayed recognition.

CCDT offers additional advantages, including ease of digital adaptation, making it suitable for remote electronic health (e-Health) interventions [5]. These interventions have the potential to empower adults over fifty, reduce clinic visit costs [35], and gather extensive longitudinal and cohort data for the application of machine learning techniques in early diagnosis [36]. Paradoxically, despite its user-friendliness, CCDT diagnostic potential remains underutilized in clinical practice [31].

More generally, while considerable research efforts are dedicated to biomarkers, relatively few have been directed to comprehending cognitive trajectories. These trajectories are fundamental for distinguishing between healthy and pathological aging at an early stage [14].

### Objectives of the study

This study is part of an ongoing project focused on exploring web-based tasks’ potential for tracking individual cognitive trajectories and providing tools for helping to sensitively identifying individuals at risk of Mild Cognitive Impairment (MCI) and dementia. Our first, primary objective is understanding cognitive trajectories across different ages. Contrasting ability which are differently modulated by age and dual-tasking requirements seems a promising way for the early differentiation between healthy and pathological aging. Specifically, we examined how aging affects cognitive performance in the context of dual-tasking. To capture individual variability in dual task cost, we tested participants aged between 50 and 90 years by using a dual-task involving two different cognitive domains (namely, memory and attention), a newly devised self-administered cognitive screening, a measure of cognitive reserve [37] and a questionnaire on subjective memory decline.

In summary, the study aims to achieve the following objectives, listed below starting from the broader ones:

• Assess the usability and feasibility of online cognitive assessments for older adults.
• Examine the relationship between memory performance, cognitive reserve, and subjective memory complaints.
• Investigate how age, individual cognitive reserve, and subjective memory complaints influence CCDT cost.
• Ascertain whether memory dual-tasking cost (DTC) within our sample is better conceptualized as a continuous or categorical variable, potentially suggestive of a subgroup displaying early (prodromal) cognitive decline.

To assess the actual usability of the tasks and feasibility of this approach, we also administered an ad hoc questionnaire with simple questions investigating to what extent participants experienced difficulties due to the online/computer-based modality.

We expected overall mnestic accuracy for visual images to worsen with age, with a relatively constant cost [5]. We further expected a protective effect of cognitive reserve on both baseline and dual-task performance, with a potential reduction in cost for persons with high cognitive reserve. Finally, we expected age-related worsening, especially in the dual-task, to be greater for those participants who complained the most about subjective memory problems.

In the case undiagnosed individuals in the prodromal phase of dementia were present in our sample we thought this subset could be leading to a discontinuity in the memory test and questionnaire scores [5]. We expected that, if present, this discontinuity, could be detected through a taxometric analysis, combining both objective and subjective memory measures.

## Methods

### Participants

500 Italian-speaking adult volunteers aged between 50 and 90 years were recruited by members of the research laboratory by word of mouth. They were unaware of the hypotheses and aims of the study. Initial contact could occur via phone or in-person, based on individual availability. During this first contact, participants were provided with an overview of the study. Those expressing their willingness to participate received clarification on exclusion criteria. Exclusion criteria were uncorrected deficits in vision and hearing, neurological pathologies or psychiatric disorders, alcohol abuse or drug history, confirmed diagnosis of MCI or dementia. Among the 440 volunteers who agreed to participate, 38 were excluded based on medical history. Subsequently, each eligible participant was sent an email containing an informed consent form, general instructions, and a link to participate in the study. Sixty-five participants did not complete all the tests and therefore were excluded. The final sample encompassed 337 volunteers aged between 50 and 89 (202 females). Data were collected confidentially and then anonymized before group analysis. Data collection started in July 2021 and lasted for 6 months. Data for the Memo and auto-GEMS tasks from a subgroup of 252 participants from the same sample (selected on the basis of mnestic accuracy) were included in a previous study [5]. Informed consent was obtained from all subjects involved in the study. The study was conducted in accordance with the Declaration of Helsinki and approved by the Institutional Ethics Committee of Department of General Psychology, University of Padova (protocol code 3744, date of approval 1/10/2020). Upon entering the initial screen of the experiment, participants were provided (for a second time) with the informed consent document. At the bottom of the page, participants were required to confirm their understanding and willingness to participate by selecting either “I accept and continue” or “I refuse and close”. If a participant declined the terms, a farewell message was displayed. Participants did not receive any compensation for their participation.

### Software and materiasls

We programmed the experimental protocol using HTML (Hyper Text Markup Language), CSS (Cascading Style Sheets) and jsPsych, an open source JavaScript library developed specifically as a framework for web-based psychological experiments [38]. We extended JsPsych capabilities by means of the plugin "jspsych-psychopshysics" which allowed for more accurate timing for visual and auditory stimuli [39]. JsPsych is a front-end JavaScript library that runs entirely on the participant’s computer. Through this methodology it is possible to remotely measure accuracy and reaction times for audio-visual stimuli with laboratory precision [40–42]. To make the protocol available through internet, we uploaded the experimental material on a webserver with a JATOS instance installed [43]. JATOS is an open source, cross-platform web application with a graphical user interface that simplifies the management of data and links to the experiment. Each participant received the same access link. The link allowed only one access based on the IP address of the computer. This allowed unique data storage for each participant. The webserver that hosted the experiment and the associated secure database were located at the Department of General Psychology, University of Padua. To participate in the study participants did not need any ad hoc hardware nor software but simply a computer (including mouse, keyboard, and loudspeakers) with internet access. To avoid unforeseen issues with the rendering of the experimental webpage, we limited the execution of the experiment to three browsers (Chrome, Firefox, Edge) which were intensively tested by the experimenters beforehand. The test battery is available at OSF repository.

### Experimental design and procedure

The experiment consisted in a set of self-administered online tests and questionnaires. Demographic information was collected after the acceptance of informed consent. Data collection started with a self-administered online cognitive screening (auto-GEMS, Pucci et al., in prep [44], adapted from a recent paper and pencil open source screening [45]), and a short online measure of Cognitive Reserve (CR) (adapted from Cognitive Reserve Index Questionnaire [46]). The memory task (Memo) included one single-task block and a second dual-task block with concomitant Continuous Performance Test (CPT) [47]. The data collection ended with two self-report questionnaires, the Memory Complaint Questionnaire (MAC-Q) [48] and a newly devised User Autonomy Questionnaire (available in the S1 Checklist). We used the outcome of the latter to discard from analysis those participants who were helped or distracted by others during the testing phase but also, more in general, to quantify the degree of “external” help needed by participants (e.g. some had no personal mail address or needed help to launch the task).

A detailed description of the experiment was provided to each participant via e-mail along with the instruction to perform the task in a sufficiently illuminated, silent and comfortable environment. If the participant was not sufficiently familiar with the computer, instructions explicitly allowed a caregiver to help preparing the set-up, which included a mouse (or a trackpad), a keyboard and loudspeakers. Participants were informed that they could interrupt the experimental procedure at any time and withdraw their consent to the use of their data. The caregiver was not allowed to help participants during the test.

### Global Examination of Mental State

The self-administered cognitive screening (auto GEMS) was designed to provide a fast and reliable measure of global cognition in approximately 10 min. It consisted in a computerized adaptation [44] derived from a recent paper and pencil open source screening called Global Examination of Mental State (GEMS) [45] and its telephonic version Tele-Global Examination of Mental State (Tele-GEMS) [49]. In the online adaptation we used, the participant responded autonomously by using a mouse and a keyboard and the scoring was automated. The auto GEMS battery consisted of a combination of closed questions, multiple choice, and structured tests measuring spatial and temporal orientation, short and long-term memory, spatial skills, verbal skills, executive functions. There were in total 10 subtests, each of them providing a score from 0 to 10 for a total of 100 points. The online demo version of the experiment, which incorporates the auto-GEMS [50], can be accessed from the OSF repository linked to this study. We corrected the auto-GEMS scores based on the correlation with the CR score, as suggested by the authors of the original battery [45, 49] as opposed to standard correction for age and education only [51, 52]. The details of correlation with CR and the equation for auto-GEMS correction are provided at the end of the Methods section.

### Memory task

For the Memo task we contrasted the single-task with the dual-task condition. Each of the two blocks consisted of a memorisation and a recognition phase of images. During the memorization phase, participants were instructed to memorize a stream of 15 black and white images depicting inanimate objects sequentially presented in the centre of the screen for 5 seconds each. Simultaneously, an auditory stream of letters (A, B, C, D, and X) was played. There were no additional visual stimuli displayed on the screen other than the images during either the single-task or dual-task conditions.

We were careful in ensuring that the single-task and dual-task conditions were as similar as possible, as previously described by Contemori et al. in 2022 [5]. Therefore, the single- and the dual-task conditions only differed for the task requirement whereas the stimuli were identical. In each block, there were 45 auditory letters (3 targets), separated by a stimulus onset asynchrony of 1.6 sec.

In the single-task condition, participants were instructed only to memorize the images to be later recognized and disregard the auditory letters. In the dual-task condition, they were additionally required to pay attention to the auditory letters and press the spacebar whenever they heard the target stimulus "X", as in the auditory version of the CPT [53, 54]. In this second, dual-task condition, participants were not required to prioritize one task over the other.

The reason for including the auditory letter stream in both the single-task and dual-task conditions was to prevent the introduction of potential confounding variables by adding extra stimuli in only one of the two conditions.

All images were selected from a database specifically created for memory studies that contains 50 different categories of objects. Within each category, multiple similar images were available. The database included an estimate of the similarity between images belonging to the same category that resulted from a previous standardisation [55, 56]. For each of the target images we selected 3 foils with different degrees of similarity with the target (high, medium or low). In this way the degree of similarity between target and foils was kept constant within each trial. Images were randomly shuffled between participants and counterbalanced across conditions.

**Fig 1:**
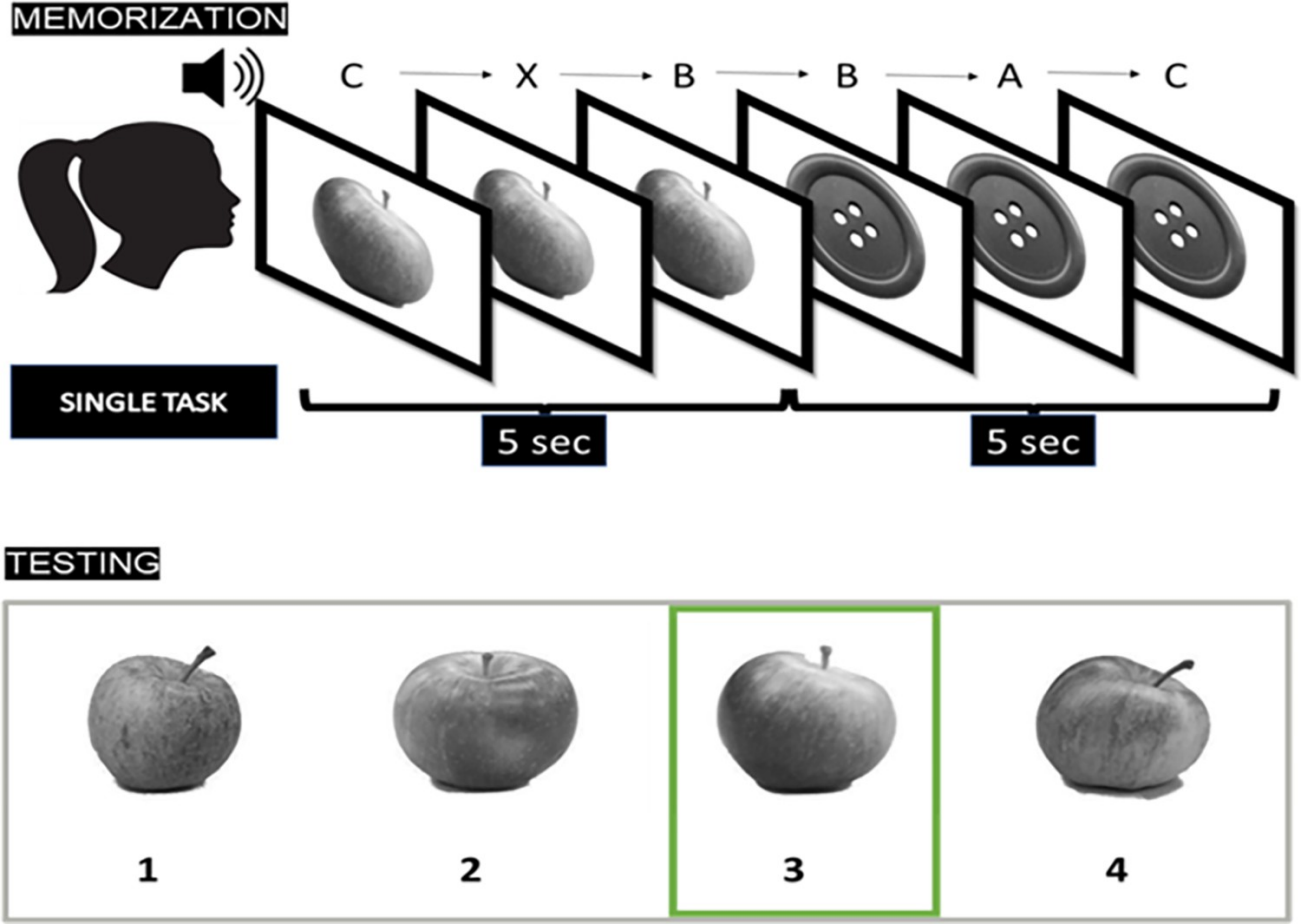
A representative set of trials. During the encoding phase 15 images were shown for 5 seconds each while a stream of auditory letters was played (each for a duration of 1.6 sec, 45 letters in total). In the single-task condition participants had to ignore the sounds. In the dual-task condition, participants had to press a key each time the letter "X" was presented.

In the recognition phase four different images belonging to the same object category (e.g., an apple, see Fig 1) were simultaneously presented. The order of the recognition trials and the position of the target among the four alternatives were randomized. Participants were explicitly asked to select the most plausible stimulus (no time limit). The single-task condition was preceded by a short practice session (3 target items). The single-task condition was always administered first, followed by the dual-task condition. This sequencing was intended to gradually introduce the task in increasing order of complexity. Having older participants to start with the more complex dual-task condition could indeed lead to confusion, resulting in atypical performance during first block of trials. While this approach may have potentially reduced the DTC due to a learning effect, it’s important to note that the same order was maintained for all participants. This consistency ensured that the sequencing did not impact how the DTC changed with age or other variables under investigation. To avoid familiarization with the stimuli, each target category was presented only once during the whole session.

The outcome of the Memo corresponded to the accuracy in the forced recognition task (chance level 25%). At trial level it was represented as a binary variable, with ’1’ indicating a correct response and ’0’ denoting an incorrect response for each of the 15 responses in each of the two blocks. For the Continuous Performance Test (CPT), a total of 45 items were presented, consisting of 3 targets and 42 distractors. Also in this case the scoring system was binary, assigning a score of ’1’ for hits or correct rejections and ’0’ for omissions or false alarms. To ensure fairness and prevent bias favouring participants more familiar with keyboards, a wide response window was employed. This was facilitated by the design, ensuring that two target stimuli could never be presented consecutively.

A response was considered correct if the response key was pressed within 3 seconds from target onset. Responses occurring within 100 ms from target onset were categorized as false alarms, along with responses occurring after more than 3000 ms from target onset.

### Online adaptation of the Cognitive Reserve Index Questionnaire (CR)

To evaluate Cognitive Reserve, we employed a short, online adaptation (CR) of the Cognitive Reserve Index Questionnaire (CRIq) [46]. The CRIq quantifies the influence of all three cognitive reserve proxies over a life span: education, working activity, and leisure time activities. The questionnaire’s design was adapted for online self-administration: the total number of items was reduced, but the score assigned to each section was kept consistent to the original CRIq by proportionally increasing the weight of the items. This allowed us to preserve a similar composed scoring range as the original version (see Nucci et al., 2012 [46]).

### Memory Complaint Questionnaire

The MAC-Q is a very short (six questions) questionnaire generating a score (range 7–35) that quantifies the degree of memory complaints. The format of the MAC-Q targets age-related changes in that a participant is asked to rate their current mnestic abilities relative to the respondent’s own baseline at the age of twenty. The first five items of the MAC-Q focus on specific contexts which older persons frequently report as troublesome regarding age-associated memory decline. The last item of the MAC-Q is a global item, pertaining to overall memory decline, which is given more weight than the other five items [48]. All MAC-Q items are rated on a 5-point Likert scale (1 Much better now, 2 Somewhat better now, 3 About the same, 4 Somewhat poorer now, 5 Much poorer now). The higher the composite score, the higher the perceived memory decline.

### User Autonomy Questionnaire

At the end of the testing user autonomy and test usability was assessed by a newly devised 8-items self-assessment questionnaire. Each item in the questionnaire, detailed in Table 2 of the Results section, explored various aspects of the participant’s performance during the experimental session through a yes/no forced-choice format.

The questionnaire generates a binary variable for each item. An affirmative response to items Q1 and Q4 indicates a high degree of autonomy, whereas an affirmative response to the remaining six questions indexes a low degree of autonomy. In our study, we analysed each item individually using descriptive statistics, without calculating a composite score.

### Data analysis

Data analysis was performed in R [57]. First, we calculated descriptive statistics for all relevant variables in this study. Second, we explored pairwise relationships between variables, by means of a Pearson correlation matrix. Significance testing for these correlations was performed with a significance threshold of α < 0.05. The DTC reported in the correlation matrix was calculated by subtracting for each participant the average accuracy in the Memo single-task from the average accuracy in the Memo dual-task, with higher cost corresponding to lower negative values.

Thirdly, we analysed memory data from Memo using a Generalized Logit-Linear Mixed Model (GLMM). The dependent variable in this model was the binomial outcome reflecting the accuracy of individual trials for each subject. To evaluate overall significance, we performed an omnibus test employing the Type-III Wald chi-square test with the "Anova" function from the "CAR" package [58]. When necessary, Benjamini–Hochberg corrected post-hoc comparisons were performed by means of the “glht” function from the “multcomp” package [59]. Since both main effects and the interaction were examined in the model, the continuous variables were centered and rescaled to avoid multicollinearity issues [60]. We included the (cognitive) Load as a within-subject factor with two levels (single vs. dual), and we also included as continuous between-subject variables the scores at auto-GEMS-c (auto-GEMS corrected for CR), MAC-Q, CR, and the participants’ Age. Moreover, we specified the participant as a random effect in the model thereby assuming a by-subject variation in the intercept for the Memo accuracy.

Finally, for determining whether the memory decline is more appropriately conceptualized and assessed in a categorical or a dimensional manner we applied the comparison curve fit index (CCFI [61, 62]) and investigated the profile of three combined memory scores (two objective, Memo single-task and DTC, and one subjective, the MAC-Q). The CCFI profile provides insights into whether memory decline is better characterized as a categorical or dimensional construct, thereby contributing to a better understanding of cognitive aging and its mechanisms. Multiple taxometric indexes can be used to compute CCFIs, as a form of consistency testing, we evaluated CCFIs from three standardly reported taxometric indexes: MAMBAC, MAXEIG (maximum eigenvalue), and L-MODE (latent mode) and averaged them together [61]. CCFI is computed as the ratio of the degree of misfit for the observed data to a dimensional population compared to a categorical population. CCFI values less than 0.45 evidencing support for a dimensional model, values greater than 0.55 evidencing support for a categorical model, and values in between indicating an ambiguous outcome. The code used to generate the analysis and figure can be found in the OSF repository associated with the study.

### Auto-GEMS correction

**Fig 2:**
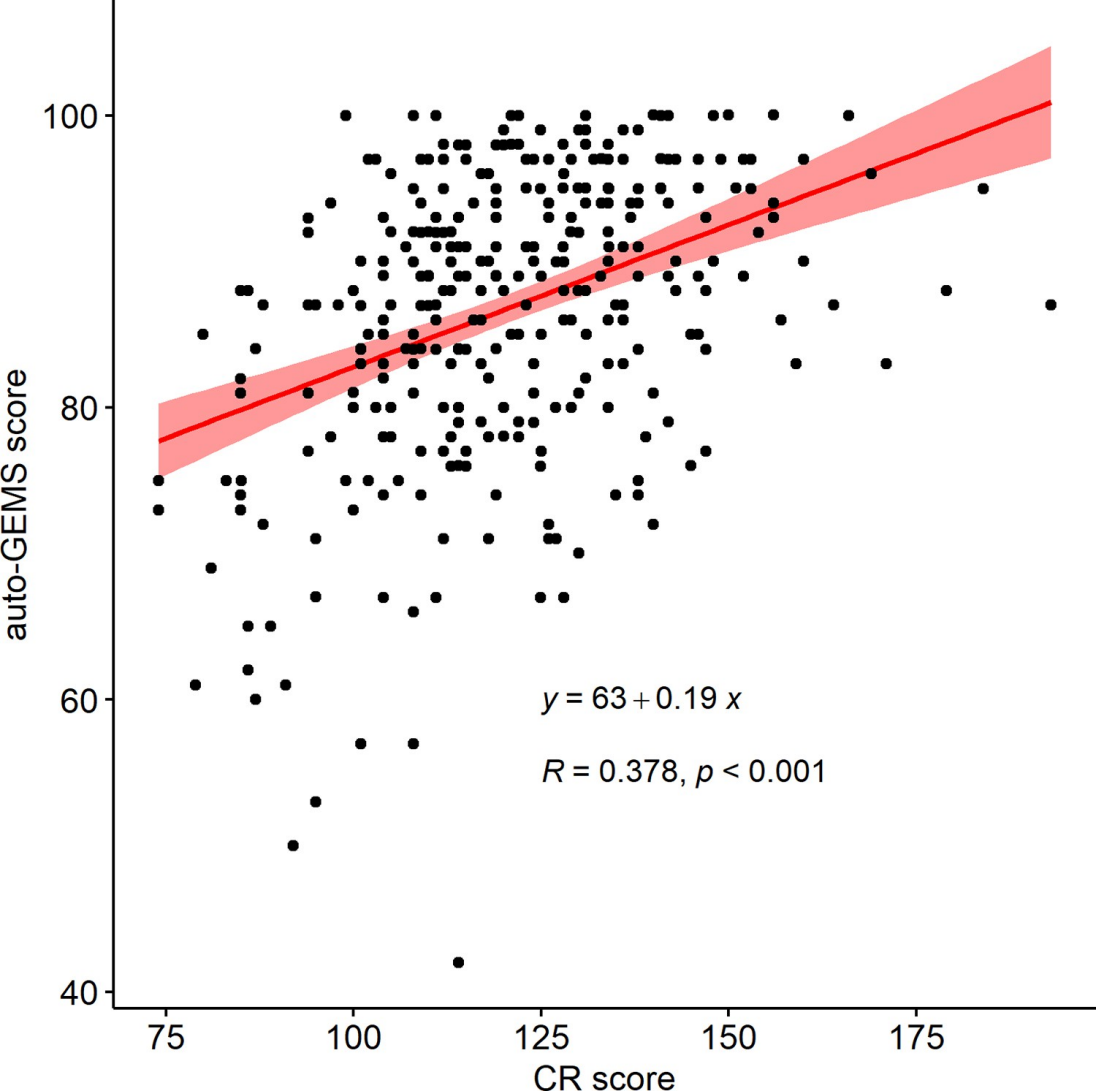
Correlation plot between remote global examination of mental state (auto-GEMS) and cognitive reserve index (CR) score. Bands represent 95° confidence intervals.


To account for between subjects’ demographic differences, we regressed-out from the auto-GEMS scores the influence of CR. The correlation between auto-GEMS and CR scores (Fig 2) revealed a significant positive relationship (R = 0.38, p< 0.001) described by the eq 1. The corrected score (auto-GEMS-c) was calculated by subtracting from the individual raw auto-GEMS scores the output of eq 1. Descriptive statistics for the auto-GEMS-c are reported in Table 1.

y=63+0.19X

**Table 1:** Descriptive statistics for the uncorrected (upper row) and corrected (lower row) auto-GEMS scores.

	Median	Range	Mean	Std	Kurtosis	Skewness
auto-GEMS	88	42–100	86.8	9.809	4.671	-1.119
auto-GEMS-c	1.83	-43.51–17.415	0	9.081	4.431	-0.967

Eq 1. Regression between cognitive screening performance (auto-GEMS) and cognitive reserve index (CR) scores.

## Results

### User Autonomy Questionnaire

The responses provided to the User Autonomy questionnaire (Table 2) show that 72.7% of the participants were able to complete the whole battery at the best of their abilities, that 8.3% where interrupted or distracted (Q2) while only 2.4% received suggestions in at least one answer (Q8). Most of the participants (55.8%) performed the test in complete autonomy (Q4), while 21.4% had a person opening the web browser and connecting to the test web page for them, and 22.9% received clarification about some of the instructions.

**Table 2:** Positive responses (percentage) to the User Autonomy Questionnaire (YES-NO answer).

Q1	**I have carried out the task to the best of my ability.**	**72.7%**
Q2	I have been interrupted or distracted during the task.	8.3%
Q3	I have carried out the task quickly, without focusing on my answers.	6.8%
Q4	I have carried out the task autonomously from start to finish.	55.8%
Q5	A person has opened the email for me and then I have carried out the task autonomously.	21.4%
Q6	I have carried out the task answering the questions autonomously, but a person helped me to use the mouse and the keyboard.	22.9%
Q7	I have carried out the task with a person who explained some questions to me.	9.8%
Q8	I have carried out the task with a person who suggested some of the answers to me.	2.4%

In addition, items Q1 and Q4, concerning autonomy in task execution, correlated with both Age (-) and auto-GEMS-c (+). On the contrary, items Q6, Q7, and Q8, concerning receiving external aids, showed a significant correlation with both Age (+) and auto-GEMS score (-). As shown in Table 3 no other correlations were significant.

**Table 3:** Correlations between the User Autonomy Questionnaire individual items, Age, and the auto-GEMS-c score.

		t	df	p-value	R
Q1	*Age*	-2.307	335	0.022*	-0.125
	*auto-GEMS-c*	4.229	335	<0.001***	0.225
Q2	*Age*	*0*.*506*	*335*	*0*.*613*	*0*.*028*
	*auto-GEMS-c*	*-1*.*573*	*335*	*0*.*117*	*-0*.*086*
Q3	*Age*	*-1*.*060*	*335*	*0*.*290*	*-0*.*058*
	*auto-GEMS-c*	*0*.*076*	*335*	*0*.*939*	*0*.*004*
Q4	*Age*	*-7*.*015*	*335*	*<0*.001***	*-0*.*358*
	*auto-GEMS-c*	*3*.*559*	*335*	*<0*.001***	*0*.*191*
Q5	*Age*	*1*.*256*	*335*	*0*.*210*	*0*.*068*
	*auto-GEMS-c*	*-0*.*532*	*335*	*0*.*595*	*-0*.*029*
Q6	*Age*	*12*.*908*	*335*	*<0*.001***	*0*.*576*
	*auto-GEMS-c*	*-6*.*648*	*335*	*<0*.001***	*-0*.*341*
Q7	*Age*	*9*.*132*	*335*	*<0*.001***	*0*.*446*
	*auto-GEMS-c*	*-7*.*83*	*335*	*<0*.001***	*-0*.*393*
Q8	*Age*	*3*.*026*	*335*	*0*.003**	*0*.*163*
	*auto-GEMS-c*	*-3*.*546*	*335*	*<0*.001***	*-0*.*191*

A high degree of autonomy is indicated by affirmative responses to items Q1 and Q4, while affirmative responses to the remaining six questions indicate a low degree of autonomy

* P ≤ 0.05

** P ≤ 0.01

*** P ≤ 0.001, ns P > 0.05.

### Descriptive statistics

**Fig 3:**
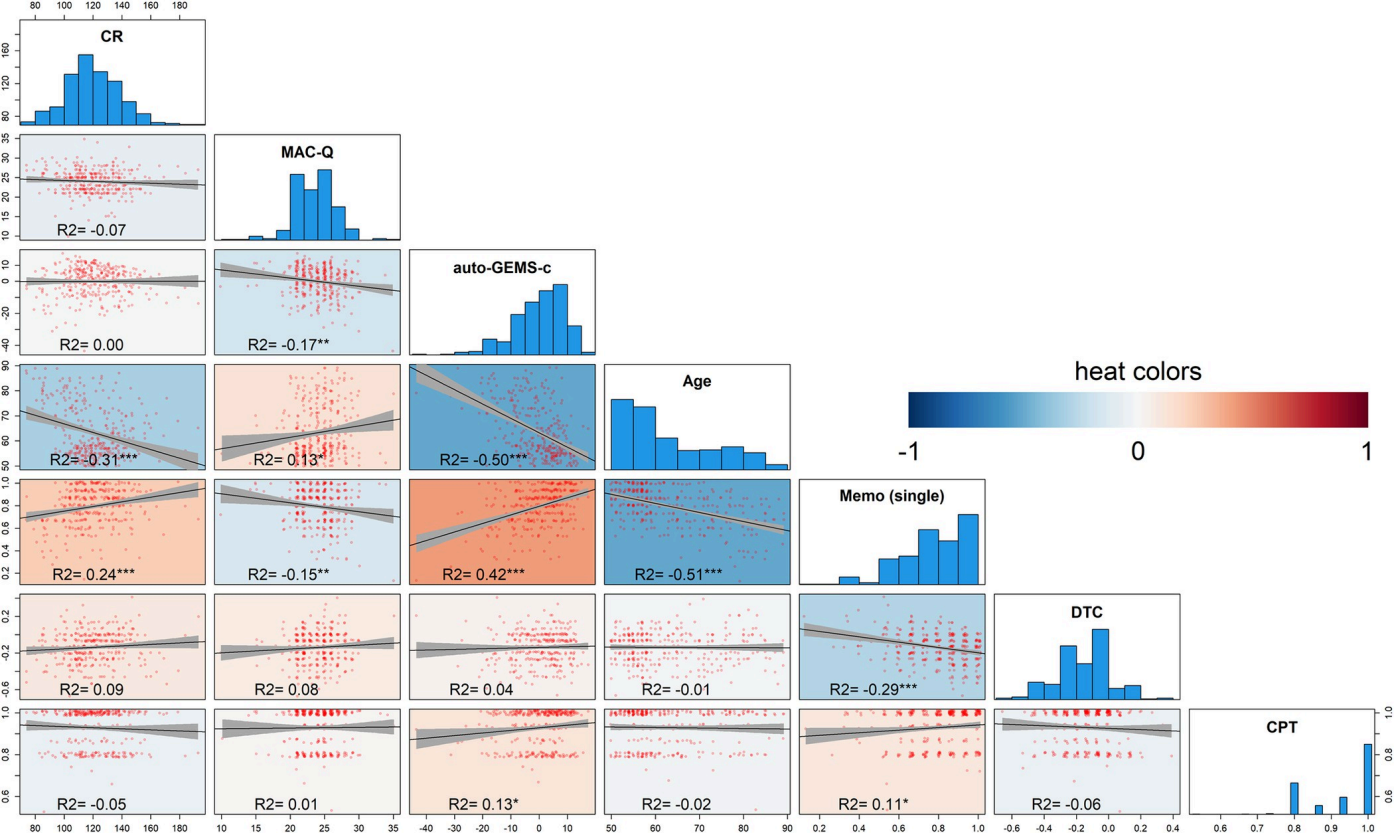
Scatterplot matrix, with jittered bivariate scatter plots below the diagonal and histograms on the diagonal (see Table 5). Pearson correlation coefficients and linear regression fits with C.I. 95% are reported. The colour gradient, ranging from blue (negative) to red (positive), illustrates the magnitude of correlation. Note the significant negative relationship between auto-GEMS-c and Age and between memory complaints (MAC-Q, increasing with Age) and objective performance both in auto-GEMS-c and in Memo single-task but not with DTC.

We found no obvious outlier but the probability distribution for the measured variables was not symmetric. Age and CR data were found to be skewed to the left (see the histogram in the diagonal in Fig 3), while Age, MAC-Q, auto-GEMS-c, and Memo scores were found to be skewed to the right. We did not find noticeable floor or ceiling effects, except in the CPT, where the average performance was 93% correct. Dataset descriptive statistics are shown in Table 4.

**Table 4:** Descriptive statistics.

	Median	Range	Mean	Std	Kurtosis	Skewness
CR	119	74–193	120.6	19.033	3.592	0.349
Schooling	13	5–25	12.91	4.925	2.138	0.006
MAC-Q	24	10–35	24.04	3.053	4.987	-0.313
auto-GEMS-c	0.202	-4.791–1.918	0	1	4.431	-0.967
Age	59	50–89	63.26	10.401	2.410	0.773
Memo single	0.8	0.133–1	0.794	0.164	3.644	-0.883
Memo dual	0.667	0.133–1	0.655	0.199	2.514	-0.360
DTC	-0.133	-0.66–0.4	-0.139	0.168	3.153	-0.188
CPT	1	0.533–1	0.929	0.089	2.826	-0.896

Reported variables are cognitive reserve (CR), years of schooling, memory complaint questionnaire (MAC-Q), cognitive screening (auto-GEMS-c) corrected score, Age, Memo single-task accuracy, Memo dual-task accuracy, Memo dual-task cost (DTC), and accuracy in the Memo secondary task (CPT). For the DTC, a more negative value corresponds to a higher cost.

**Table 5:** Correlation matrix for cognitive reserve index (CR), memory complaint questionnaire (MAC-Q), cognitive screening (auto-GEMS-c), Age, Memo single-task accuracy, dual task cost (DTC), and Continuous Performance Test (CPT) accuracy.

	CR	MAC-Q	auto-GEMS	Age	Memo single	DTC	CPT
*CR*	1						
*MAC-Q*	-0.071	1					
*auto-GEMS-c*	0	-0.170**	1				
*Age*	-0.314***	0.134*	-0.501***	1			
*Memo single*	0.240***	-0.145**	0.420***	-0.506***	1		
*DTC*	0.093	0.079	0.038	-0.011	-0.287***	1	
*CPT*	-0.054	0.011	0.126*	-0.023	0.109	-0.057	1

* P ≤ 0.05

** P ≤ 0.01

*** P ≤ 0.001, ns P > 0.05.

### Correlation matrix

The correlation matrix (Fig 3) allows an exhaustive graphical overview of the relationship among different indexes. CRI negatively correlated with Age (R = -0.314, p< 0.001) and positively with Memo single-task (R = 0.240, p< 0.001). Conversely, the MAC-Q negatively correlated with auto-GEMS-c (R = -0.170, p = 0.002), Age (R = 0.134, p = 0.014), and Memo single-task (R = -0.145, p = 0.008). Moreover, auto-GEMS-c strongly correlated with Memo single-task (R = 0.420, p< 0.001), consistently with the presence of immediate and delayed memory tests in the auto-GEMS battery. Interestingly, Age was also strongly negatively correlated with Memo single-task (R = -0.506, p< 0.001) and auto-GEMS-c (R = -0.501, p< 0.001) but not with DTC (R = -0.011, p = 0.838) nor with CPT (R = -0.023, p = 0.677). DTC only correlated with Memo single-task (R = -0.287, p< 0.001), while CPT mildly correlated with auto-GEMS-c (R = 0.126, p = 0.021) and Memo single-task (R = 0.109, p = 0.045). These correlations are further illustrated by the scatterplots in Fig 3.

As expected, the CPT/sustained attention task was overall smoothly performed as participants had on average 93% of correct answers. There was no significant correlation between CPT performance and participants’ age (R = -0.023, p = 0.676) nor between CPT performance and the concurrent Memo dual-task (R = 0.042, p = 0.440). However, CPT interference led to a decreased accuracy by 0.138 in Memo dual task vs Memo single-task. This indicates that, on average, the likelihood of correctly identifying visual targets in the forced-choice task decreased by 13.8% when participants were previously also asked to attend to the audio stream of letters from the CPT.

In the CPT, out of the 337 participants, 41 committed at least one false alarm, and 260 achieved at least one hit (out of 3). Interestingly, the sub-group made by 77 participants who did not achieve any hits still showed a dual-tasking cost of -0.115. This suggests that even those participants who were unsuccessful in identifying any of the three target stimuli likely exerted effort in performing the secondary task.

**Fig 4:**
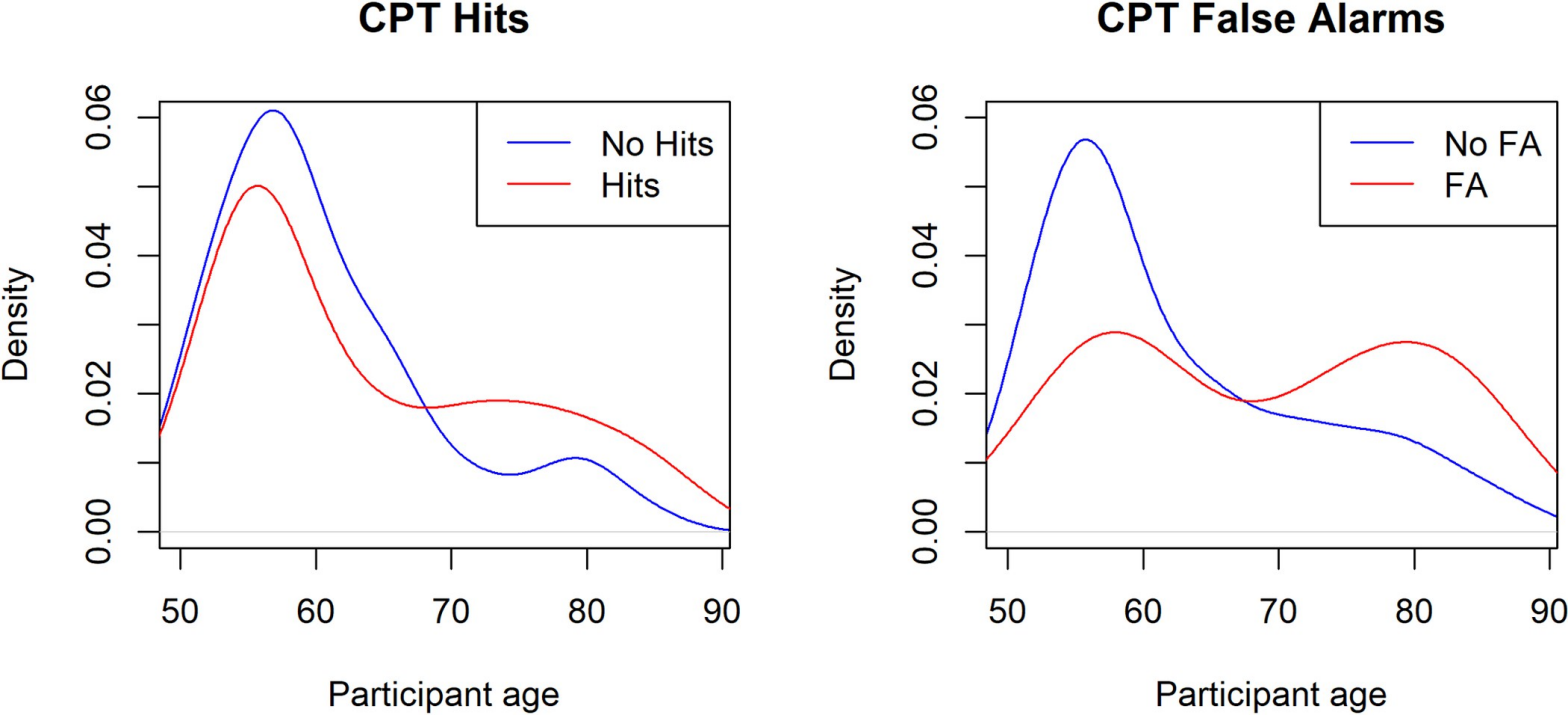
Density plots illustrating the age distribution of participants based on the number of Hits and False Alarms (FA) in the auditory Continuous Performance Test (CPT). Panel A on the left displays the density plot for CPT Hits. The blue curve represents participants with zero Hits, while the red curve represents those with at least one Hit. Panel B on the right showcases the density plot for CPT FA. Here, the blue curve corresponds to participants with no FA, and the red curve represents those with at least one FA.

Furthermore, the Welch Two Sample t-test comparing the memory cost between those who achieved at least one hit and those who did not achieve any hits in the CPT did not indicate a significant difference (t = -1.283, df = 111.62, p = 0.202). We also conducted an asymptotic two-sided two-sample Kolmogorov-Smirnov test to compare the age distribution of individuals who had at least one hit in the CPT with those who had no hits. This test yielded a significant outcome, with a D = 0.194 and a p = 0.023. This suggests a likely age difference between individuals with and without hits. Contrary to intuitive expectations, as depicted in Fig 4, the density of individuals with zero hits decreased more with increasing age compared to those achieving at least one hit.

Similarly, when comparing the age distributions of individuals who committed false alarms in the CPT with those who did not, a significant difference was observed (D = 0.280 with a p = 0.007). The density of individuals who committed at least one false alarm decreased less with age, in contrast to those with zero false alarms.

### Memo task

The analysis of deviance with the Type III Wald chi-square tests for the Memo score showed a significant main effect of Age (Chisq = 35.245, df = 1, p< .001) with the Memo accuracy decreasing with participant’s age increasing and a significant main effect of auto-GEMS-c (Chisq = 16.443, df = 1, p< .001) with the Memo performance increasing together with auto-GEMS score. Also, a significant main effect of (cognitive) Load was detected (Chisq = 162.878, df = 1, p< .001) with the Single-task resulting in a higher score with respect to the Dual-task (estimate = 0.751, se = 0.059, z = 12.76, p < .001). We also found a significant main effect of CR (Chisq = 8.082, df = 1, p = 0.004) with the mnestic score decreasing with participant’s CR decreasing. In short, the task effectively induced all the expected main effects. Among the two-way interactions, Age by Load was significant (Chisq = 6.297, df = 1, p = 0.012) with the difference between single and dual Memo decreasing as age increased. Also, the interaction Age by CR was significant (Chisq = 5.028, df = 1, p = 0.025), as the difference between high and low CR decreased as age increased. We found a significant three-way interaction, auto-GEMS-c by Load by CR (Chisq = 5.085, df = 1, p = 0.024). We found one significant four-way interaction, auto-GEMS-c by Load by MAC-Q by CR (Chisq = 5.227, df = 1, p = 0.022). All other interactions were not significant.

**Fig 5:**
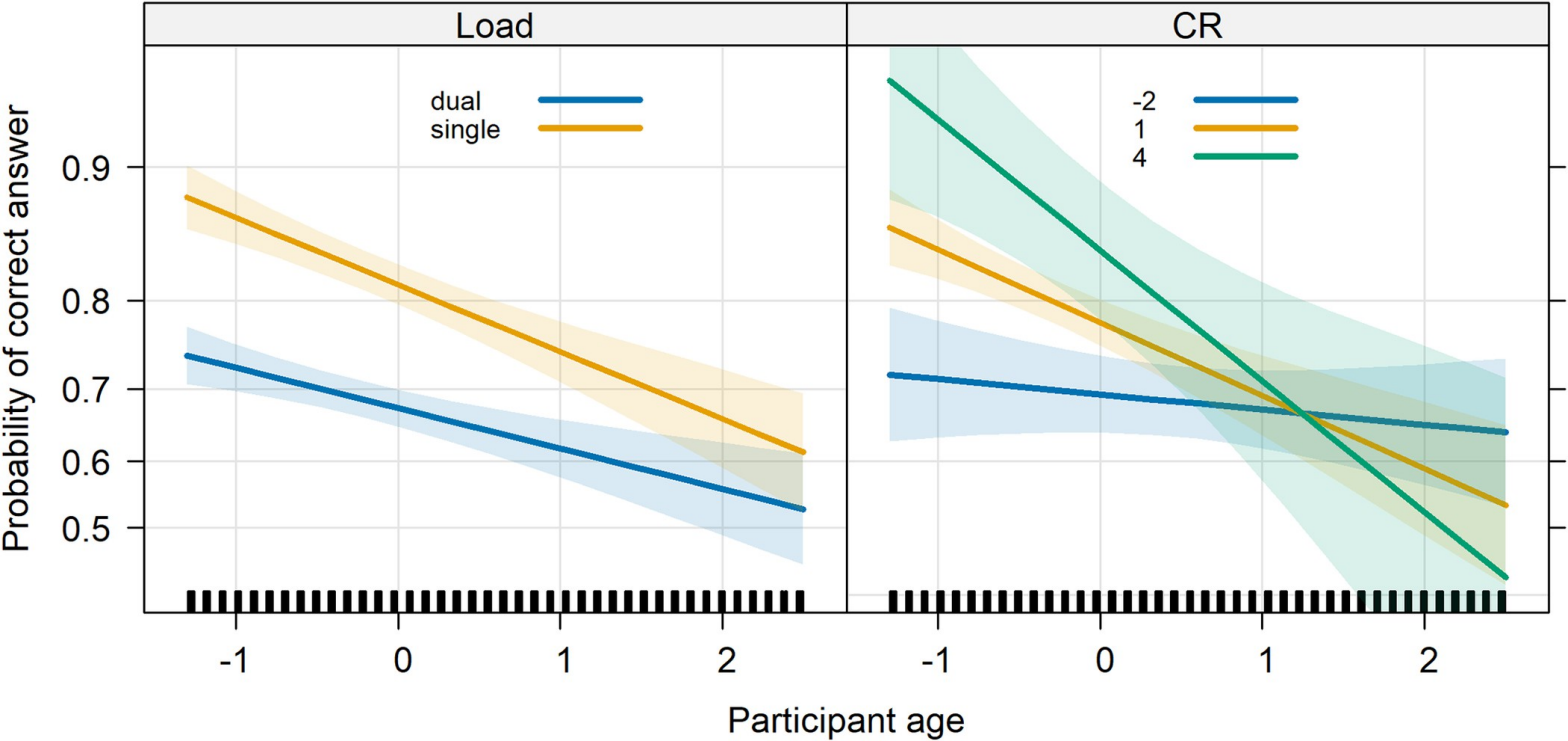
Left panel, Memo accuracy as a function of Age and Load (single or dual). Right panel, Memo accuracy as a function of CR, and Age. Bands represent 95° confidence intervals.

**Fig 6:**
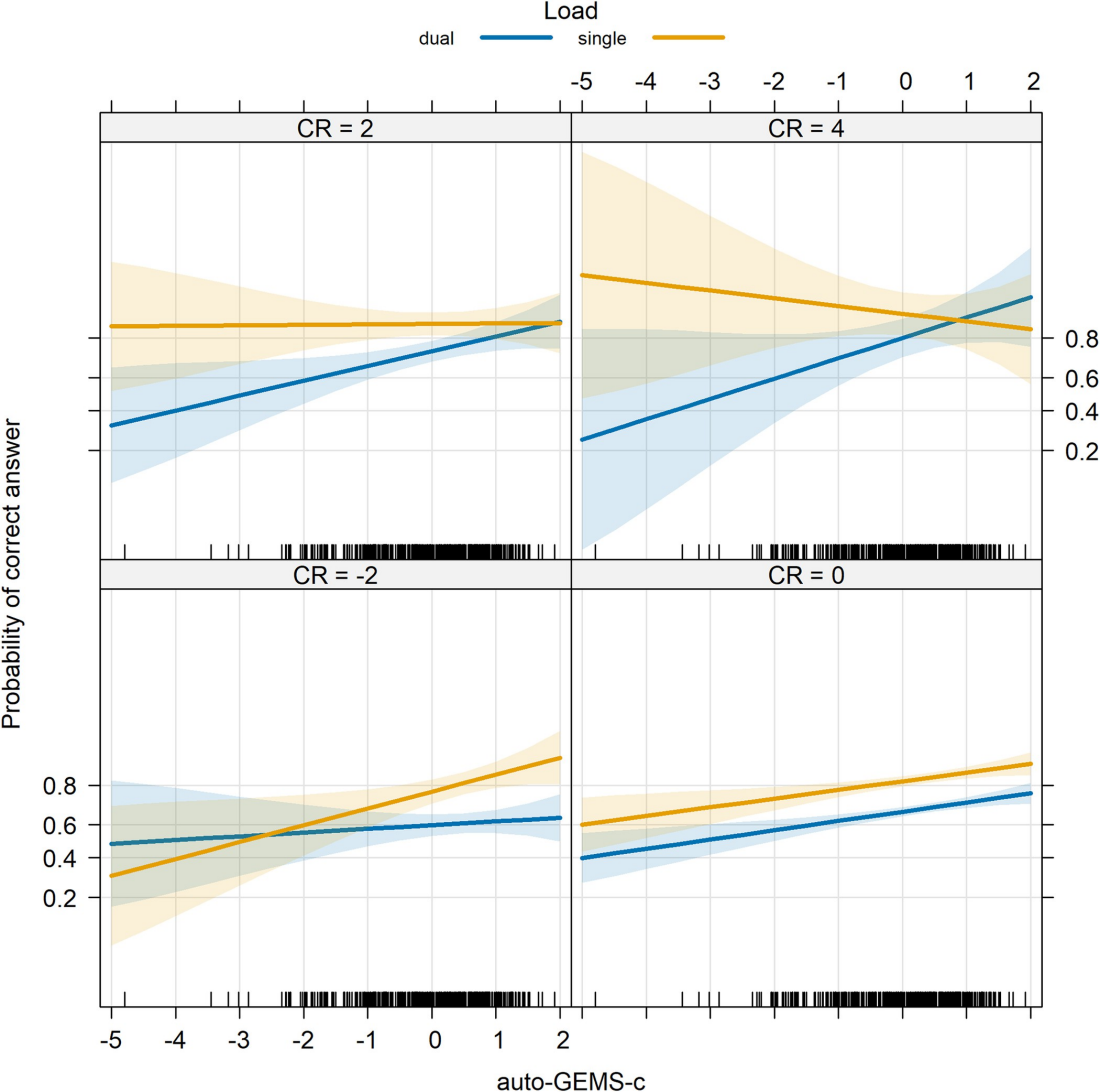
**Memo accuracy as a function of auto-GEMS-c, Load (single or dual), and CR.** Bands represent 95° confidence intervals.

**Fig 7:**
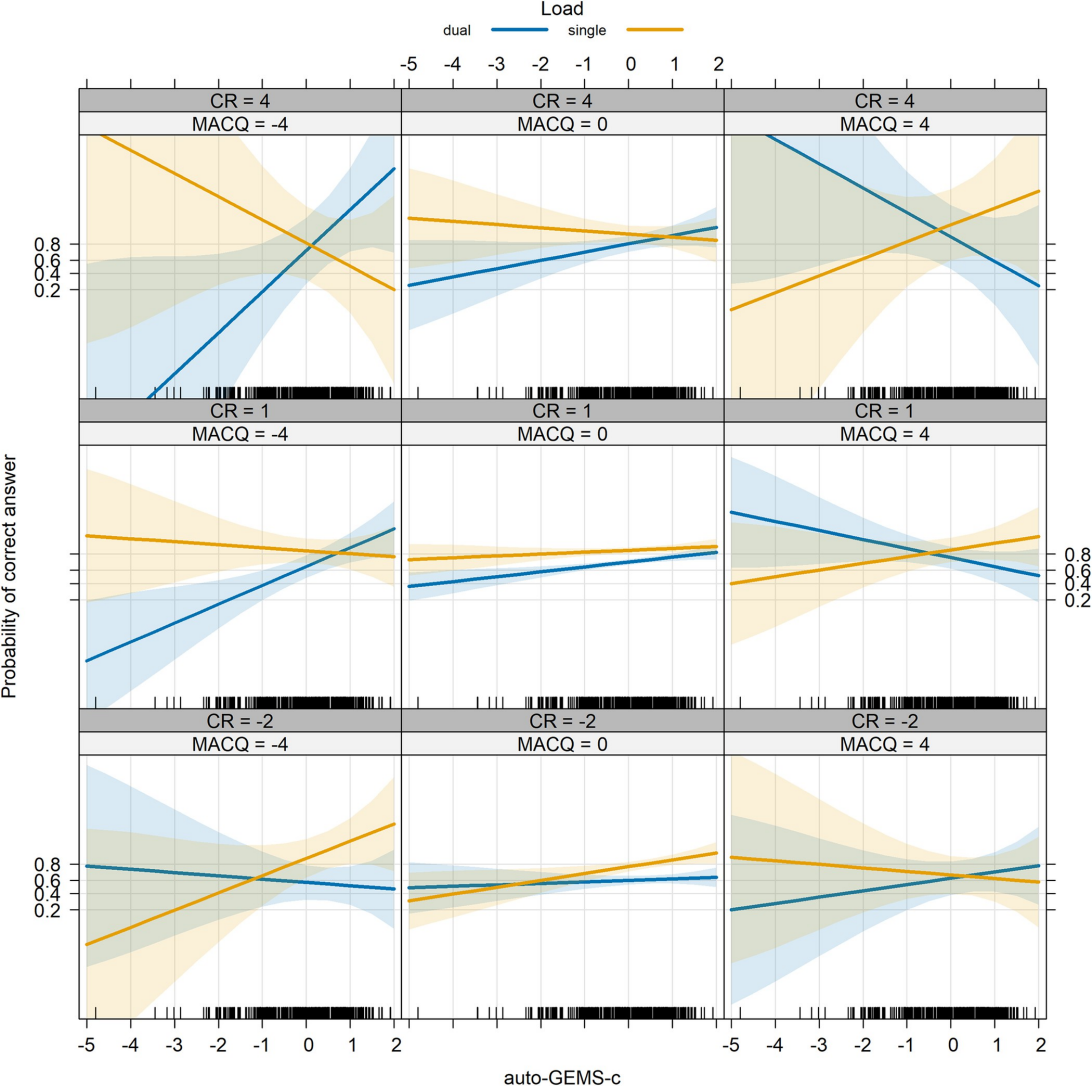
Memo accuracy as a function of auto-GEMS-c, Load (single or dual), MAC-Q, and CR. Bands represent 95° confidence intervals.

In short, Age, Load, auto-GEMS-c and CR significantly predicted mnestic performance. Notably, the dual-tasking cost exhibited a decrease, rather than an increase, with age, as illustrated in Fig 5. The significant three-way interaction is illustrated in Fig 6, while the significant four-way interaction is illustrated in Fig 7. The complete list of model fixed effects is given in Table 6.

**Table 6:** Analysis of deviance with the Type III Wald chi-square tests for the Memo task (accuracy).

	Chisq	Df	Pr(>Chisq)
(Intercept)	481.817	1	<0.001***
Age	35.245	1	<0.001***
auto-GEMS-c	16.443	1	<0.001***
Load	162.878	1	<0.001***
MAC-Q	0.036	1	0.850
CR	8.082	1	0.004**
Age:auto-GEMS-c	0.017	1	0.895
Age:Load	6.297	1	0.012*
auto-GEMS-c:Load	0.051	1	0.822
Age:MAC-Q	3.144	1	0.076
auto-GEMS-c:MAC-Q	1.425	1	0.233
Load:MAC-Q	2.651	1	0.103
Age:CR	5.028	1	0.025*
auto-GEMS-c:CR	0.070	1	0.792
Load:CR	0.390	1	0.532
MAC-Q:CR	0.309	1	0.578
Age:auto-GEMS-c:Load	1.334	1	0.248
Age:auto-GEMS-c:MAC-Q	1.396	1	0.237
Age:Load:MAC-Q	0.611	1	0.435
auto-GEMS-c:Load:MAC-Q	3.342	1	0.067
Age:auto-GEMS-c:CR	0.539	1	0.463
Age:Load:CR	0.479	1	0.489
auto-GEMS-c:Load:CR	5.085	1	0.024*
Age:MAC-Q:CR	0.667	1	0.414
auto-GEMS-c:MAC-Q:CR	0.022	1	0.883
Load:MAC-Q:CR	0.262	1	0.609
Age:auto-GEMS-c:Load:MAC-Q	0.152	1	0.697
Age:auto-GEMS-c:Load:CR	0.352	1	0.553
Age:auto-GEMS-c:MAC-Q:CR	0.948	1	0.330
Age:Load:MAC-Q:CR	3.644	1	0.056
auto-GEMS-c:Load:MAC-Q:CR	5.227	1	0.022*
Age:auto-GEMS-c:Load:MAC-Q:CR	1.078	1	0.299

* P ≤ 0.05

** P ≤ 0.01

*** P ≤ 0.001, ns P > 0.05.

### Taxometric analysis

To determine whether the memory decline is more appropriately conceptualized in a categorical or a dimensional manner we applied the CCFI to three combined memory scores, two objective (Memo single-task and DTC), and one subjective (MAC-Q).

The MAMBAC CCFI profile (0.41) was in favor of a dimensional structure in relation to memory decline (0.41), while the MAXEIG (0.59) and L-MODE (0.59) profiles propended for a continuous structure. The combined average of the three CCFI profiles (0.53) was therefore inconclusive and failed to confirm the existence of a neat subdivision between healthy aging and prodromal pathological aging in our sample.

## Discussion

In this study, we describe the performance collected in a group of more than 300 healthy adults, aged between 50 and 90 years, by using self-administered, online cognitive tests. By using a multiple-choice format, we collected accuracy in the recognition of images representing inanimate objects. A single-task condition was contrasted with a dual-task condition which required to concurrent pay attention to an auditory stream of letters. We also tested whether certain individual factors such as global cognitive functioning, cognitive reserve, the presence of subjective memory complain, or cognitive load modulate visuo-mnestic performance differently at different ages. Results for the Memo task reveal a decline in visuo-mnestic performance associated with lower global mental state scores and lower cognitive reserve, but also with increasing age and cognitive load. Notably, the difference between the Memo single- and dual-task decreased rather than increased, for older participants (see Fig 5). The cost remained consistent across the age cohort, aligning with the findings of our previous study with a complementary approach focused on performance in two different dual-task conditions [5]. One might argue that the outcome of GLMM, with data analyzed at the individual trial level, is more sensitive. However, the crucial point is that neither of the two analyses revealed an increase in cost. Importantly, we did not find any significant correlation between the dual-task cost (DTC) and the Continuous Performance Test (CPT), nor between CPT performance and age. This result dismisses the idea that older participants present a specific trade-off between their primary and secondary tasks, which could account for the reduced cost observed in the Memo task.

Interestingly, participants who failed in the secondary task (omitting to respond to all target letters) still exhibited, on average, a cost in the primary task, indicating their attempt to engage in both tasks. Furthermore, when comparing the age distribution of those who hit at least one target with those who did not, a distinct pattern emerged: younger participants were more likely to miss targets, while older individuals were more prone to commit false positives. This pattern aligns with the hypothesis of an inhibition deficit in older age, suggesting that older adults may struggle more than young adults overcoming dominant responses or ignoring distracting information [63–65]. In short, the findings of the Memo task show that a potential marker of healthy aging is the lack of increased cost when coupling a visuo-mnestic task with a divided attention manipulation. It should be noted that the fixed order within the mnestic task, where all participants completed a single-task block before the dual-task block, may have mitigated the overall cost across the entire sample. Despite this, the cost persisted across all age groups, indicating that the absence of age-related cost escalation cannot be attributed to this factor.

Furthermore, we observed that as cognitive reserve (CR) increased, performance on the Memo task improved. This effect was modulated by age as it was observed in younger participants but not in older ones (see Fig 5). This may indicate that the protective effect of cognitive reserve weakens with age. Moreover, the difference between single and dual Memo (i.e., DTC) was larger for individuals with lower CR score and lower cognitive performance (auto-GEMS-c). Due to lower performance in the single Memo, they performed similarly independently of the load level.

Our results, although unexpected, fit within a reasonably delineated framework. Previous studies utilizing the MCDT have consistently demonstrated an age-related decline in dual-task ability in the motor domain, but seldom in the cognitive domain. Moreover, empirical evidence is scant for the specific domain of CCDT based on visual memory.

A meta-analysis, encompassing 34 studies (including both CCDT and MCDT) conducted between 1981 and 2003, reveals a distinct age-related impairment in dual-tasking. However, the critical moderating variable seems to be the nature of the task. Tasks involving significant controlled processing or a motor component exhibit more pronounced dual-task impairment compared to simpler tasks relying on automatic processing [66]. For instance, the cost is evident in both the motor and secondary cognitive tasks when the latter involves processing tasks such as arithmetic calculations [3].

Although the auditory version of the CPT used in our study was intentionally simple, the mnemonic task was inherently complex regardless of age. In 2003, Verhaeghen et al. conducted a meta-analysis, finding that the impact of dual-task cost remains consistent across various task complexities. The study noted an age-related increase in cost when calculated based on reaction time but not in logit-transformed accuracy [67]. A lack of correlation between age and cost has been similarly documented in a few other studies, examining both reaction time and accuracy [4, 5, 68–71]. Hence, we suggest that the absence of a clear correlation between interference and age, may be due to the specific cognitive processes engaged by the task, rather than merely its difficulty level.

It is well established that there are age-related breakdowns in working memory capacity and attentional control—the ability to stay on task and avoid cognitive distractions. This is illustrated by age-related declines in performance on divided attention tasks (see Watson et al., 2011, for a review [72]) and the ability to handle interruptions [65].

In our study, we posit that attending to auditory stimuli and pressing the button when necessary "interrupts" the memorization process. While working memory impairments in older adults may be linked to heightened attention to distractors (a suppression deficit), impairments induced by interruptions do not seem to be clearly mediated by age-related increases in attention to interrupters [65]. Working memory can be characterised as a system for the simultaneous information storage (maintenance) and information processing (manipulation) [73]. Notably, our study using an auditory continuous performance task as a secondary task did not result in an age-related increase in cost; in contrast, other tasks requiring higher working memory involvement exhibit such an increase. For example, Jaroslawska et al. (2021) reported a more pronounced decline in processing performance in older adults across various task domains compared to younger counterparts [74]. Similarly, Rhodes et al. (2019) found that introducing an arithmetic processing task during a 10-second delay disrupted the retention of serially presented letters, with disruption magnitude increasing with increasing age (18 to 81). Importantly, task demands were adjusted beforehand to ensure age differences did not reflect baseline disparities in single-task performance [75]. Notably, age-related differences in working memory dual-tasking are not attributed to encoding speed limitations [76]. Verhaeghen and Zhang (2013) tested younger and older adults with a modified version of the N-back task. They found a dual-task cost and an interference cost, as well as a large age effect. However, neither the dual-task nor the interference effect appeared to be sensitive to age [68]. Heidemann et al. (2022) tested two groups, one of younger and one of older adults, with a dual retrieval task involving the retrieval of two responses, one vocal and one keypress, from a single cue. They also demonstrated that dual memory retrieval appears to remain relatively stable across different age groups [69].

We could therefore conclude that across several empirical investigations the cost did not increase with age in working memory tasks. However, the relationship between age and the cost of dual memory tasks is more complex, as demonstrated by Riby and colleagues in their 2004 study. In this research, both young and older adults were instructed to retrieve previously learned associates (episodic retrieval) or overlearned category members (semantic retrieval) under conditions of single or working-memory load. Cued recall and recognition procedures were employed to assess performance. The findings suggest that the cost of dual-tasking, in terms of proportional effects, remained consistent across age groups for semantic retrieval. However, a noticeable age-related increase in cost was observed for episodic retrieval. Nonetheless, the impact of age on recognition was smaller compared to cued recall [71]. To summarize earlier findings, an age-related increase in cost appears in motor and processing tasks (i.e., information transformation), while attentional and semantic memory tasks (i.e., information maintenance) do not clearly exhibit this relationship. Our present findings support the notion that the presence of an age-related cost increase depends on the specific cognitive domain under investigation. We found no age-related increase in cost for familiarity-based memory. We chose a familiarity-based test as a strategic measure to overcome ceiling effects among the young and floor effects among the elderly. Opting for a recollection memory test could have potentially yielded divergent results [77]. Moreover, this methodological choice allows for the identification of individuals undergoing prodromal stages of Mild Cognitive Impairment (MCI). While older adults demonstrate more pronounced impairment in recollection compared to familiarity testing [16], individuals with MCI or early Alzheimer’s disease (AD) typically show worse performance when recognition is based on familiarity [17–20]. The age-related recollection deficit is thought to be driven by inefficient episodic encoding [78]. Despite being challenging even for younger participants, the cost in the familiarity task remained stable across different age groups [5]. Interindividual variability in cost is primarily attributed to individual predisposition and possibly pathological conditions deviating from normal aging [5]. Previous research indicates a chronological progression of cognitive impairments in Alzheimer’s disease, linking early neurodegeneration of the perirhinal/anterolateral entorhinal cortex to compromised familiarity for items requiring discrimination as viewpoint-invariant conjunctive entities [79].

It is crucial to note that the results may differ when testing samples consisting of various age groups or continuous age cohorts. The significance of the sampling strategy is highlighted by Sebastián and Mediavilla’s (2017) study, specifically focusing on dual-task cost in a memory task [70]. They discovered that differences in dual-task coordination were evident only when the sample was divided into two broad age groups, not when narrower age groups were considered. This suggests that comparing older and younger individuals in separate groups magnifies differences that are not apparent when analysing the continuous effect of age, as undertaken in our present study.

Regarding MAC-Q, correlational analysis indicates that subjective complaints increase with age and decrease with auto-GEMS-c. Additionally, no main effect on the Memo task was identified, but MAC-Q played a significant role in a four-way interaction involving auto-GEMS-c, Load, and CR. Specifically, individuals who expressed more complaints and had lower auto-GEMS-c scores tended to exhibit lower mnemonic performance in the single Memo. Surprisingly, however, they performed well in the dual Memo, in contrast to those who complained less. This difference was more pronounced for those with lower CR. This unexpected result may be clarified by the heightened attention that individuals with high MAC-Q scores allocate to tasks they perceive as vulnerable, leading to overcompensation. This finding aligns with the notion that memory complaints impact coping behavior due to memory-related anxiety [80].

Past studies suggest that memory complaints not only mirror perceptions of past memory performance but also might serve as an early indicator of future memory performance (i.e., memory impairments) [81] although mostly in the verbal domain. However, individuals assess their memory performance not in absolute terms but in relation to what they consider typical for peers of the same age. Consequently, correlations between self-reported memory impairments and performance on standardized memory assessments vary widely, as memory complaints are linked to personality, self-efficacy, anxiety, and depression symptoms for some individuals [81, 82]. Previous research indicates that perceived memory decline predicts future depressive symptoms, whereas memory ratings do not. Depressive symptoms do not forecast future memory complaints [83]. Given the frequent association between memory complaints and depressive symptoms in older adults, both may serve as potential indicators of future cognitive decline in a possible longitudinal follow-up of the current cohort study.

The four-way interaction between age, load, MAC-Q, and CR also supports the possibility of overcompensation by older individuals with high CR and high MAC-Q (higher subjective memory complaints) that is not found in other peers and younger individuals [84, 85]. A plausible interpretation suggests that individuals perceiving memory difficulties may employ compensatory strategies, which prove more effective for those with a higher cognitive reserve, resulting in above-average performance. Despite an equal priority being assigned to both tasks, a prioritization effect similar to that achieved by varying explicit instructions [75] may manifest for those who are sensitive to memory issues and pay greater attention to their mnemonic performance.

In summary, these results demonstrate that even in a familiarity-based recognition task, considered relatively preserved in older persons, age-related effects exist, partially mitigated by high cognitive reserve.

**Fig 8:**
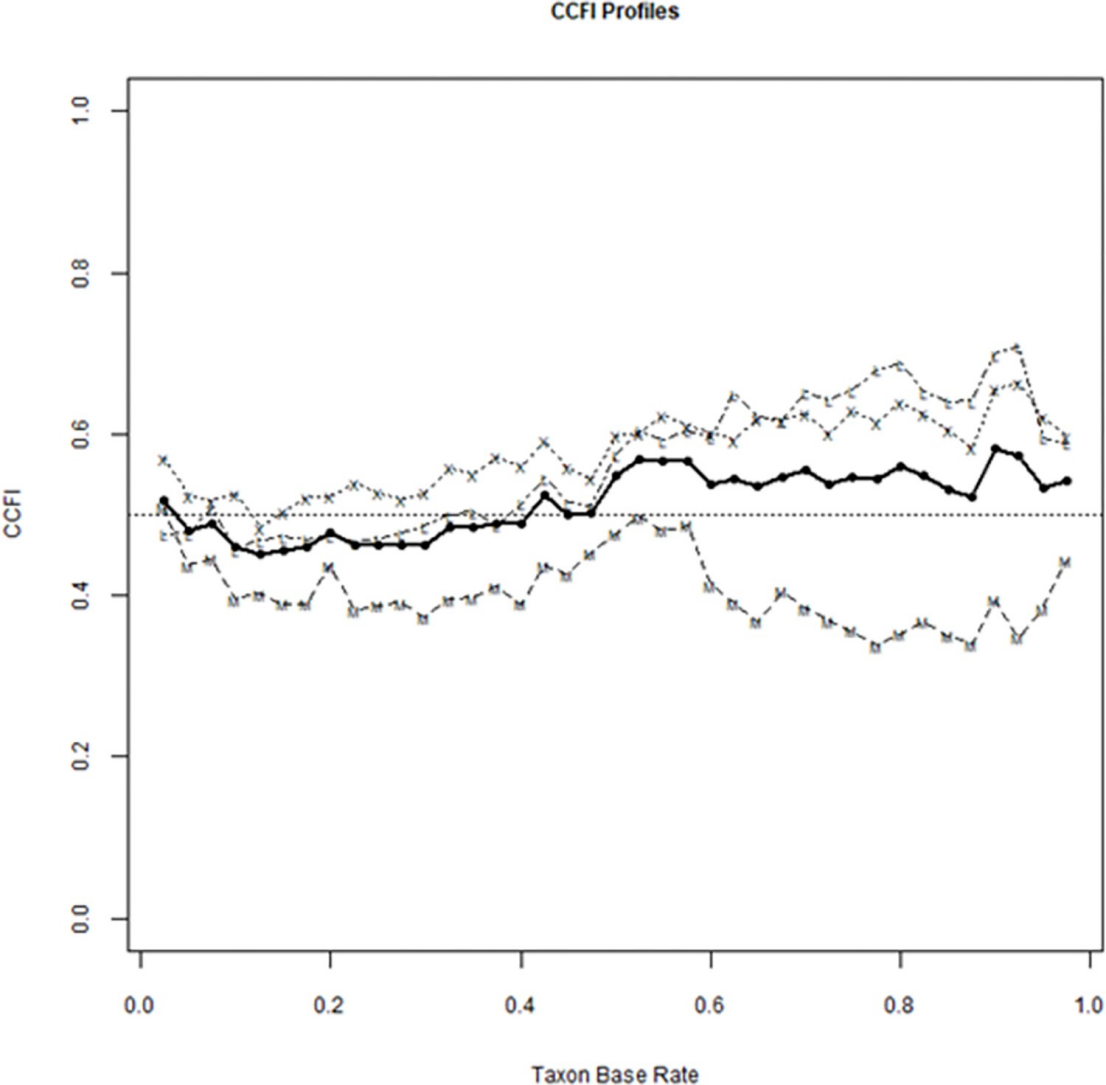
CCFI profiles from taxometric analyses of three unique sets of memory indicators, two objective—Memo single-task and DTC, and one subjective MAC-Q. M = MAMBAC, X = MAXEIG, L = L-MODE, and solid dots = the average CCFI.

An alternative explanation for conflicting outcomes in prior studies regarding dual cognitive task cost is based on differences in the tested sample selection. Discrepancies in results may be attributed to subgroups in tested samples with specific characteristics. In some ostensibly healthy samples, a subset of older adults engaged in protective cognitive activities [86] may coexist with other subgroups exhibiting prodromal MCI or dementia [5]. Taxometric analysis based on Memo single-task, DTC, and MAC-Q score could not distinguish between a dimensional and continuous structure in memory performance (see Fig 8) therefore not showing the presence of a distinct group of potentially pathological cases in our sample. In our prior research involving a similar CCDT, the grouping of multiple cost indexes enabled the detection of diverse performances at the auto-GEMS [5]. Despite this promising outcome, the inconclusive results from the current taxometric analysis suggest that relying solely on memory indices–without longitudinal data–is inadequate for identifying a subgroup with characteristics indicative of the prodromal phase of cognitive decline, as statistically anticipated in such a sizable sample [87].

Our study presents a proof of concept for a multidomain approach that combines objective and subjective scores from an online self-administered memory assessment, with the aim of developing a tool for sensitively measure cognitive trajectories. These approaches might pave the way for the early detection of cognitive impairment that can use large-scale longitudinal monitoring of healthy older patients. Ideally, such a tool should identify cases that deviate from the normal trajectory of age/education matched peers and individual performance history. Although the longitudinal multivariate approach appears promising and can account for both the differences in the timing of decline of different cognitive functions and the individual differences at baseline, a fundamental issue remains in identifying the variables (i.e., the test battery) most sensitive for performance monitoring and potential onset of decline.

Accuracy-based longitudinal tests are difficult to develop because they require careful calibration to avoid "ceiling" performance in younger people and "floor" performance in older people. Indeed, the single Memo task is easy for the youngest participants who are then tested on the dual-task to appreciate individual differences. In contrast, older participants do not show a noticeable decline in the dual-task, as do those with high MAC-Q scores. Thus, in this case, to fully characterize the mnestic profile of individuals of different ages might be necessary to collect more information from other domains.

These findings are simply an initial step in the long way leading to the validation of a clinical tool. Still, this initial step seems relevant considering the importance of the issue at stake as well as the relative gap of knowledge characterizing the combined impact of aging and cognitive load on mnestic performance. This new approach might lead to a variety of new, promising scenarios on the possibility of continuous monitoring over time, through repeated measures over a lifetime, with a rapid, self-administered instrument. It then becomes possible to compare the individual’s performance both with their history and with a normative sample of reference peers. This is particularly valuable for individuals with high CR and strong compensatory abilities who may otherwise fall into the ambiguous zone of traditional neuropsychological assessments. The non-verbal characteristics of the Memo task and the minimal differences among target and foils make it a very suitable option for reducing learning due to test-retest. Expanding the image pool would allow testing with a broader range of stimuli, enhancing the validity of repeated assessments.

Regarding auto-GEMS, our study employed version A (but version B is also available), administered once to each participant. To support repeated usage, developing additional parallel versions, especially for memory-related subtests, is imperative. This would better address learning and familiarity effects in test-retest scenarios. Expanding both the Memo and auto-GEMS tests is pivotal for transitioning from a cohort study to a longitudinal application.

The trend towards self-administered computerized testing is supported by older participants’ improved digital skills, with about 70% completing tests independently, a level once deemed utopian. Those most reliant on caregiver assistance, roughly 20%, tended to be the oldest or those with lower auto-GEMS scores. However, explicit suggestions for answers were rare, occurring in only 2.4% of cases. Thus, caregiver presence during testing does not skew results but rather aids in mitigating variability in technological proficiency, maintaining data quality. As digital literacy among older adults increases, computerized cognitive testing will likely become more prevalent and feasible.

Though these findings represent an initial step towards employing more sensitive cognitive tests, longitudinal studies will be necessary, potentially excluding MCI or dementia cases. Many studies track indices from health to pathology, but we seek subtler markers, possibly non-pathological in isolation. Testing MCI may not offer significant insights, as they often exhibit cognitive impairments reflecting widespread issues. While evidence from other cognitive domains [23] show that MCI are impaired at dual-tasking (as in any other difficult task), it remains unclear how early these deficits manifest.

## Conclusion

In our study, we investigated how 300 adults aged 50 to 90 performed on visuo-mnestic tasks. We found that as age increased, there was a decline in task performance, but the costs associated with these declines remained relatively stable. Surprisingly, we observed that older individuals did not exhibit higher dual-task costs, which challenges common expectations and underscores the complexity of cognitive aging. Additionally, we examined factors such as cognitive reserve and subjective memory complaints. Our findings suggest that the protective effects of cognitive reserve diminish with age, while there may be compensatory mechanisms at play.

Furthermore, our analysis of memory indicators using taxometric methods produced inconclusive results. We did not find a clear distinction between healthy individuals and a possible subgroup showing signs of early pathological aging.

Our study is a proof-of-concept about the feasibility and clinical potential of online, self-administered multidomain tests that encompass both subjective and objective measures based on cognitive-cognitive dual-task for cognitive assessment. While promising for sensitive monitoring, challenges such as ceiling and floor effects must be addressed. Further research and refinement of these tools are essential for broader applications in cognitive health. This indicates that these tests can effectively assess an individual’s cognitive status over time and potentially identify those at risk for impairment.

## Supporting information

S1 Checklist *PLOS ONE* clinical studies checklist. (PDF)
